# Valproic acid triggers increased mitochondrial biogenesis in POLG-deficient fibroblasts

**DOI:** 10.1016/j.ymgme.2014.03.006

**Published:** 2014-05

**Authors:** Kamil S. Sitarz, Hannah R. Elliott, Betül S. Karaman, Caroline Relton, Patrick F. Chinnery, Rita Horvath

**Affiliations:** a Institute of Genetic Medicine, Newcastle University, Central Parkway, Newcastle upon Tyne NE1 3BZ UK; b MRC Integrative Epidemiology Unit, Oakfield House, University of Bristol, Bristol BS8 2BN, UK; c Nijmegen Center for Mitochondrial Disorders, Radboud University, Nijmegen Medical Center, Nijmegen, The Netherlands

**Keywords:** VPA, valproic acid/sodium valproate, mtDNA, mitochondrial DNA, *POLG*, polymerase γ, AHS, Alpers–Huttenlocher syndrome (OMIM: 203700), MIRAS, mitochondrial recessive ataxia syndrome, SANDO, sensory ataxia, neuropathy, dysarthria, ophthalmoplegia (OMIM: 607459), CPEO, chronic progressive external ophthalmoplegia (OMIM: 157640), MEM, minimal essential medium, FBS, fetal bovine serum, NEAA, non-essential amino acids, BSA, bovine serum albumin, PVDF, polyvinylidene difluoride, cDNA, complementary DNA, HRP, horseradish peroxidase, Valproic acid (VPA), Toxicity, mtDNA, POLG, Methylation

## Abstract

Valproic acid (VPA) is a widely used antiepileptic drug and also prescribed to treat migraine, chronic headache and bipolar disorder. Although it is usually well tolerated, a severe hepatotoxic reaction has been repeatedly reported after VPA administration. A profound toxic reaction on administration of VPA has been observed in several patients carrying *POLG* mutations, and heterozygous genetic variation in *POLG* has been strongly associated with VPA-induced liver toxicity.

Here we studied the effect of VPA in fibroblasts of five patients carrying pathogenic mutations in the *POLG* gene. VPA administration caused a significant increase in the expression of *POLG* and several regulators of mitochondrial biogenesis. It was further supported by elevated mtDNA copy numbers. The effect of VPA on mitochondrial biogenesis was observed in both control and patient cell lines, but the capacity of mutant *POLG* to increase the expression of mitochondrial genes and to increase mtDNA copy numbers was less effective. No evidence of substantive differences in DNA methylation across the genome was observed between *POLG* mutated patients and controls. Given the marked perturbation of gene expression observed in the cell lines studied, we conclude that altered DNA methylation is unlikely to make a major contribution to POLG-mediated VPA toxicity. Our data provide experimental evidence that VPA triggers increased mitochondrial biogenesis by altering the expression of several mitochondrial genes; however, the capacity of POLG-deficient liver cells to address the increased metabolic rate caused by VPA administration is significantly impaired.

## Highlights

• VPA caused the increased expression of *POLG* and other mitochondrial proteins *in vitro*.
• Elevated mtDNA copy number confirmed the effect of VPA on mitochondrial biogenesis.
• POLG-deficient cells cannot address the increased metabolic rate triggered by VPA.
• Altered DNA methylation is unlikely to contribute to POLG-mediated VPA toxicity.

## 1 Introduction

Valproic acid (2-propyl-pentanoic acid, VPA) is a widely prescribed antiepileptic drug used in several different types of epilepsy, but also in other neurological conditions such as bipolar disease, migraine prophylaxis, alcohol and other sedative-hypnotic withdrawal syndromes and occasionally for chronic pain [1–3]. Although VPA is usually well tolerated, severe hepatotoxic reaction including Reye-like liver failure and VPA-induced hyperammonemic encephalopathy have been reported over the last decades [2]. VPA toxicity can be fatal, particularly in children < 2 years of age, those with developmental delay or those with metabolic disorders, such as deficiencies of the mitochondrial respiratory chain [4,5]. The severity and frequently unexpected occurrence of VPA toxicity has caused lot of debate about the application of this widely used medication and initiated major interest in solving the “mystery” of mechanisms behind VPA toxicity. Although different pathways have been suggested, no single cause has been identified to explain this reaction.

Mutations in the mitochondrial DNA (mtDNA) polymerase γ (*POLG*) cause variable neurological presentations such as Alpers–Huttenlocher syndrome (AHS) [6], ataxia neuropathy spectrum including the phenotypes previously referred to as mitochondrial recessive ataxia syndrome (MIRAS), sensory ataxia, neuropathy, dysarthria, ophthalmoplegia (SANDO) [7] and chronic progressive external ophthalmoplegia (CPEO) plus parkinsonism [8]. A profound toxic reaction on administration of VPA has been observed in several patients carrying *POLG* mutations, and this observation provided a potential clue for unveiling, at least in part, the mitochondrial mechanisms behind VPA toxicity. Children with AHS due to autosomal recessive *POLG* mutations have a particular predisposition to VPA toxicity. AHS is a childhood encephalopathy characterized by developmental delay and intractable epilepsy and liver disease [6,7]. Approximately 1/3 of AHS patients developed liver failure within 3 months of exposure to VPA [9,10]. This raises the possibility that genetic variation in *POLG* may predispose individuals to VPA-induced liver failure who may not have a recognizable phenotype like AHS [11]. In order to define the mechanism of VPA toxicity in *POLG*-related disease, we performed further *in vitro* studies including a search for an epigenetic mechanism, given the known role of VPA as a histone deacetylase (HDAC) inhibitor.

## 2 Materials and methods

### 2.1 Patient cell lines

Primary human fibroblasts were selected from five *POLG*-deficient patients (P1–P5) and three normal healthy controls (C1–C3) (Table 1). Three *POLG*-mutant cell lines (P1, P2 and P3) were derived from unrelated patients with AHS secondary to compound heterozygous *POLG* mutations: (i) P1: p.Gly737Arg and p.Ala767Asp; (ii) P2: p.Ala467Thr and p.Ser1104Phe; and (iii) P3: p.Trp748Ser and c.3600delT. The remaining two *POLG*-deficient patient lines (P4 and P5) came from unrelated individuals with MIRAS secondary to (i) compound heterozygous p.Ala467Thr and p.Trp748Ser *POLG* mutations (P4) and (ii) homozygous p.Trp748Ser *POLG* mutation (P5). This study has the relevant ethical institutional approval and written informed consent was obtained from all of the subjects involved.

### 2.2 Cell culture studies

Fibroblasts were cultured in MEM (Gibco) supplemented with 10% (v/v) FBS (Gibco) 50 U/ml penicillin and 50 μg/ml streptomycin (Gibco), 1 × MEM Vitamin Solution (Gibco), 1 mM sodium pyruvate (Gibco), 1 × NEAA (Gibco), 2 mM L-glutamine (Gibco) and 50 μg/ml uridine nanopure (Sigma). Cells were grown in tissue culture flasks (Greiner) at 37 °C in a humidified cell culture incubator containing 5% CO_2_. For both patients and controls, we used cells at similar passage numbers (below 5). To investigate the effect of VPA on control and *POLG*-affected fibroblasts, cells were cultured in media supplemented with different VPA (Sigma) concentrations (0, 2, 5, 10 and 30 mM VPA) and various time points (3, 6, 8 and 10 days). All the cells died within few days of exposure to 30 mM VPA. At the same time, no obvious morphological changes were observed with 5 mM VPA after 10 days; hence, we decided on 10 mM to be the final VPA concentration in our model system. Cell viability was assessed by daily observations of morphology and density of fibroblasts using an inverted phase contrast microscope (Leica). The cellular pellets were obtained from (i) untreated fibroblast lines and (ii) cells cultured for 10 days in 10 mM VPA-supplemented growth media.

### 2.3 Immunoblotting

Fibroblast pellets were lysed in buffer containing the following: 5% (v/v) 1 M Tris (ph 7.5), 2.6% (v/v) 5 mM NaCl (VWR), 0.04% (v/v) 0.5 M MgCl_2_ (VWR), 1% (v/v) Triton™ X-100 (Sigma) and Complete™ protease inhibitor cocktail (Roche). The lysis mixture was then centrifuged at 1000*g* for 2 min at 4 °C. Protein concentrations were determined using Bradford assay (Bio-Rad) with BSA standards. Protein samples (20 μg) were separated on 4–15% precast Tris–HCl gradient gels (Bio-Rad), transferred to polyvinylidene difluoride (PVDF) membranes (Bio-Rad) and immunoblotted with the following primary antibodies: (i) rabbit monoclonal anti-POLG (Abcam, Cat.# ab128899; 1:2000), (ii) mouse monoclonal anti-SDHA (Abcam, Cat.# ab14715; 1:2000), (iii) mouse monoclonal anti-COX2 (Abcam, Cat.# ab110258; 1:2000) and (iv) rabbit polyclonal HRP-conjugated anti-GAPDH (Santa Cruz, Cat.# sc-25778; 1:2000). Subsequently, membranes were incubated with HRP-conjugated secondary antibodies.

### 2.4 Quantification of mtDNA copy numbers

Total genomic DNA was isolated from the cellular pellets using DNeasy® Blood & Tissue Kits (Qiagen). The quantification of mtDNA copy number was performed as previously described [12].

### 2.5 Gene expression studies

RNA was isolated from fibroblast pellets using RNeasy® Mini Kit (Qiagen). cDNA was synthesized from isolated RNA using SuperScript™ III First-Strand Synthesis System (Invitrogen). Gene expression was detected with SYBR® Green (Bio-Rad). Data were normalized to *GAPDH* and *ACTB* and analyzed using qBase Plus software (Biogazelle). Primer sequences for investigated genes are available upon request.

### 2.6 DNA methylation studies

Genomic DNA (500 ng) was bisulphite modified using EZDNA kits (Zymo Research, CA, USA) according to manufacturer's instructions. After random ordering, 200 ng bisulphite-converted modified DNA was processed and analyzed using the Infinium HumanMethylation450 BeadChip assay (Illumina) to quantitatively determine DNA methylation status at more than 450K sites throughout the genome. Processing was performed according to manufacturer's instructions. All samples passed internal quality controls on the array, which included controls for bisulphite conversion, hybridization and staining. Data pre-processing and normalization were conducted using a previously developed analysis pipeline [13]. Briefly, methylated or unmethylated signals that were determined using 3 or fewer beads on the array were associated with a detection *P*-value equal to 1. Samples with less than 80% of probes with detection *P*-values of ≥ 0.01 were dropped from the analysis (*n* = 19849). Probes containing SNPs were removed from the data set (*n* = 17196). SNP data were obtained from the 1000 genomes project and included SNPs with allele frequencies of ≥ 5% in the European population [14]. Probes aligning to > 1 genomic region, allowing for 1 mismatch, were removed (*n* = 24707). X and Y chromosome probes were also removed from the analysis (*n* = 10578). Colour-bias adjustment was made, based on a smooth quantile normalization method developed in the R package Lumi. Background correction was applied using negative control probes on the array. Finally subset quantile normalization was utilized, with a reference quantiles set computed from Infinium I signals for each kind of probe category according to the “relation to CpG” annotations provided by Illumina.

### 2.7 Statistical analysis

For the purpose of group comparisons, statistical analysis was performed using GraphPad™ v.5 statistical software (GraphPad Software). Group comparisons were considered to be statistically non-significant (ns) if the calculated *P*-value was greater than 0.05, significant (*) with *P* = 0.01 to 0.05, very significant (**) with *P* = 0.001 to 0.01 and extremely significant (***) when the *P*-value was less than 0.001.

## 3 Results

### 3.1 Immunoblotting

**Fig. 1:**
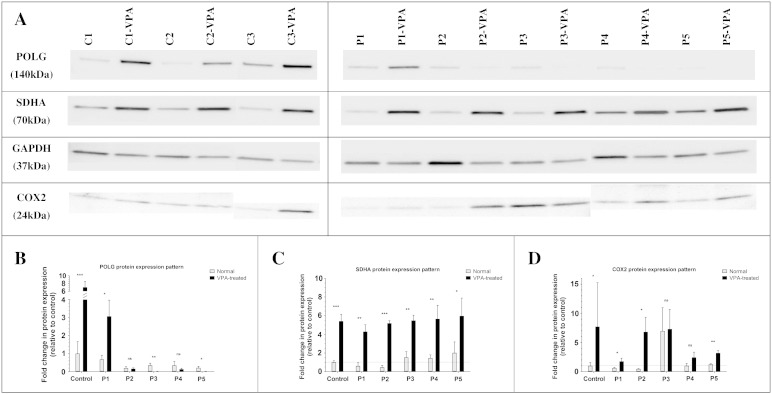
Whole cell protein was isolated from the control and patient fibroblasts cultured for 10 days in pure basal (Normal) and 10 mM VPA-supplemented (VPA-treated) media. Cell lysates at 20 μg per lane were subjected to western blotting for POLG, SDHA, COX2 and GAPDH (A). The levels of protein expression for POLG (B), SDHA (C) and COX2 (D) were normalized to GAPDH and quantified relatively to the untreated control (average of four controls). Western blot analysis is the representative of three independent biological replicates. The statistical significance of the protein expression fold changes between VPA-untreated and VPA-treated arms is indicated as follows: NS at *P*-value > 0.05; *P = 0.01 to 0.05; **P = 0.001 to 0.01; ***P < 0.001.

In order to investigate the effect of a 10-day exposure to 10 mM VPA on the expression of mitochondria-associated proteins, western blot analysis was performed on cell lysates from control and *POLG*-affected individuals to look at the levels of (i) POLG, (ii) SDHA (nuclear-encoded mitochondrial protein), (iii) COX2 (mtDNA-encoded protein) and (iv) GAPDH (loading control) (Fig. 1A). The quantification of POLG levels (Fig. 1B) revealed an unexpected 7-fold increase in its expression in the VPA-treated controls when compared to control cells not exposed to the drug. At the same time, VPA treatment resulted in an increase in POLG level in only one *POLG*-deficient line (P1), whereas in the remaining patient lines, POLG expression remained either constant (P2 and P4) or significantly reduced (P3 and P4) when compared to non-treated fibroblasts. Interestingly, the only *POLG*-mutated line, which responded to VPA treatment with an increase in POLG expression (P1), also revealed the highest POLG load of all patient lines in normal basal conditions. However, the VPA-induced increase in POLG expression in the P1 line was only of 3-fold comparing to a 7-fold change observed in the controls. The protein expression levels of SDHA, a protein that can be treated as the indicator of the mitochondrial mass, were significantly increased in all VPA-treated control and patient lines (Fig. 1C). The levels of *COX2* expression, a mitochondrial-encoded subunit of complex IV, were significantly elevated in all VPA-exposed lines, apart from P3, which however presented raised *COX2* expression load even at basal conditions in normal culture medium.

### 3.2 Quantification of mtDNA copy numbers

**Fig. 2:**
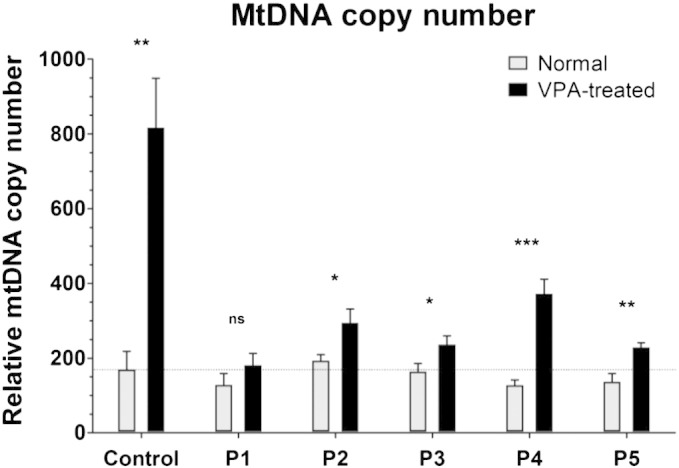
Relative mtDNA copy number in control and patient fibroblasts cultured for 10 days in pure basal (Normal) and 10 mM VPA-supplemented (VPA-treated) media. The standard deviations were derived from the three independent technical replicates. The statistical significance of the differences in mtDNA content between VPA-untreated and VPA-treated arms is indicated as follows: NS at *P*-value > 0.05; *P = 0.01 to 0.05; **P = 0.001 to 0.01; ***P < 0.001.

In an attempt to evaluate the influence of exposure to VPA on mtDNA copy number, we compared the relative mtDNA loads in fibroblasts after a 10-day culture with 10 mM VPA and without the drug, both in control and in patient lines. Significant VPA-induced increase in mtDNA copy number was observed in all investigated lines (apart from P1); however, control cells revealed a more pronounced (4-fold) elevation when compared to a maximal 2-fold increase presented by *POLG*-affected lines (P4) (Fig. 2).

### 3.3 Gene expression studies

**Fig. 3:**
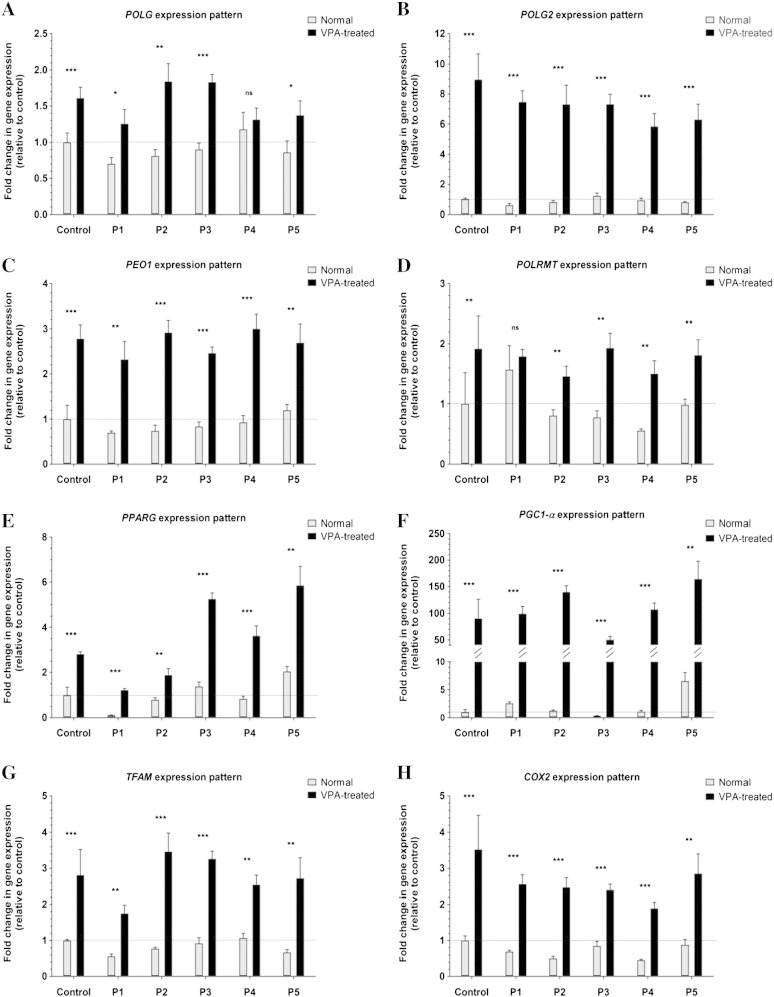
Gene expression measurements in control and patient fibroblasts cultured for 10 days in pure basal (Normal) and 10 mM VPA-supplemented (VPA-treated) media: (A) *POLG*, (B) *POLG2*, (C) *PEO1*, (D) *POLRMT*, (E) *PPARG*, (F) *PGC-1α*, (G) *TFAM* and (H) *COX2*. The standard deviations were derived from the three independent technical replicates. The statistical significance of the differences in gene expression between VPA-untreated and VPA-treated arms is indicated as follows: NS at *P*-value > 0.05; *P = 0.01 to 0.05; **P = 0.001 to 0.01; ***P < 0.001.

To investigate whether a 10-day exposure to 10 mM VPA had an effect on the expression of mitochondria-associated genes, we determined expression patterns of selected genes involved in mtDNA maintenance (*POLG*, *POLG2*, *PEO1*, *POLRMT*) (Figs. 3A, B, C and D, respectively), mitochondrial biogenesis (*PPARG, PGC-1α, TFAM*) (Figs. 3E, F and G, respectively) and OXPHOS (*COX2*) (Fig. 3H). Gene expression data revealed that treatment of both control and patient cell lines with VPA promotes a significant increase in the expression of all studied genes. In particular, the expression of *PGC-1α* which encodes a master regulator of mitochondrial biogenesis was markedly (> 50-fold) elevated in VPA-treated controls and *POLG*-affected fibroblast lines (Fig. 3F).

### 3.4 DNA methylation studies

**Fig. 4:**
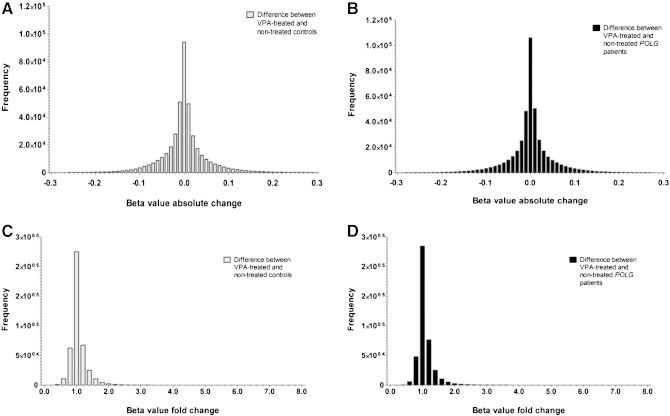
Histograms showing changes in methylation between VPA-treated and non-treated: controls (A—absolute change; C—fold change) and *POLG*-mutated patients (B—absolute change; D—fold change). All calculations are based on beta-values for 413,247 CpG sites.

To explore whether VPA treatment induced any notable changes in DNA methylation, differences in beta-values for 413,247 CpG sites distributed throughout the genome were determined between VPA-treated and non-treated (i) controls (Figs. 4A and C) and (ii) *POLG*-deficient patients (Figs. 4B and D). No major changes in overall DNA methylation patterns were present. We investigated the methylation status for genes previously assessed in terms of their expression patterns (section above) but found no significant change in CpG sites following multiple test correction (Table 2). Lastly, we compared the number of (i) all, (ii) mitochondria, (iii) liver and (iv) both mitochondria and liver-associated “top hit” CpG sites (i.e. where methylation changed with more than 20% and more than 2-fold) between VPA-treated controls and *POLG*-affected patients. No significant differences were however present between these two cohorts (Table 3).

## 4 Discussion

VPA is an 8-carbon 2-chain fatty acid, and it has been repeatedly suggested to alter fatty acid metabolism through interference with mitochondrial beta-oxidation [15]. A clear accumulation of long-chain acyl-carnitines has been shown in control fibroblasts after 2 mM VPA treatment for only 4 days [16]. Beside the competitive inhibition of beta-oxidation enzymes, the depletion of carnitine [17], coenzyme A [18] and glutathione [19] as an effect of VPA metabolism was suggested to impair lipid metabolism resulting in steatosis. Oxidative stress has been also proposed to contribute to VPA toxicity [19]. Very recently, novel targets were suggested to explain VPA-induced liver toxicity, showing that the hyperammonemia observed in children under VPA treatment may be a result of the direct inhibition of the N-acetylglutamate activity by forming valproyl-CoA, and that the reduced availability of N-acetylglutamate would impair the flux through the urea cycle and compromise the major role of this pathway in ammonia detoxification [5]. Another study highlighted that the cytotoxic action of VPA is mediated by lysosomal membrane leakiness along with reactive oxygen species formation and a decline in the mitochondrial membrane potential [3]. Although the exact metabolic pathways are not completely understood, the metabolic profiling of organic acid and amino acid metabolism suggested a potential age-related susceptibility to VPA toxicity [4].

Since the initial description of liver failure following VPA treatment in AHS patients carrying *POLG* mutations numerous, similar cases have been described, leading to a statement recommending the avoidance of valproic acid in children with a suspected *POLG*-related disease [10]. This poses a major clinical challenge due to the broad clinical spectrum and variable age of onset of disease caused by mutations in *POLG* [10]. To further complicate the scenario, heterozygous genetic variation in *POLG* has been also strongly associated with VPA-induced liver toxicity [11]. It was shown that although the role of 2 genetic variants, p.Q1236H and p.E1143G in *POLG*-related disease, is not clear, these variants may be disadvantageous in specific contexts, hypothetically such as exposure to VPA. Primary cells of *POLG* patients and controls showed severely compromised cellular proliferation when treated with VPA. Despite the observed cell death, mtDNA copy numbers did not decrease, no detectable mtDNA deletions were observed, no evidence of apoptosis was noted and β-oxidation metabolites remained within normal limits [11]. Retrospective analysis of our previous data also supports an increase in mtDNA copy numbers.

Unlike skeletal muscle and brain, the liver can proliferate in response to damage, and there is clear evidence of hepatocyte proliferation in patients with AHS. This raised the possibility that VPA compromises the regenerative capacity of the liver, thus inhibiting the endogenous capacity for liver repair in response to an external insult [11].

This being the case, why are patients with *POLG* mutations at increased risk of VPA-hepatotoxicity? It has been previously suggested that VPA inhibits histone deacetylases and alters methylation patterns and therefore may affect the regulation of gene expression by relaxing chromatin structure and facilitating access of the transcriptional machinery to the DNA [20]. In this study, we investigated whether VPA results in significant changes in gene expression or protein expression profile of mitochondrial and/or liver-specific factors, which could be due to epigenetic mechanisms such as DNA methylation. Although we agree that liver cells of POLG-deficient patients would have been more representative to study the hepatotoxic effect of VPA *in vitro*, the application of primary human hepatocyte cultures is limited, because they undergo a rapid dedifferentiation process [21]. Therefore, we performed our investigations on primary human fibroblasts.

Based on the VPA-induced liver failure in patients with *POLG*-related disease, we expected a negative effect of VPA on the expression of *POLG* and other mitochondrial genes. However, our results indicated the opposite; VPA resulted in significant increase in the expression of *POLG* and also several other mitochondrial proteins. The most striking change was a > 50-fold increase in the gene expression of *PGC-1α*, which encodes a master regulator of mitochondrial biogenesis. Since *PGC-1α* is also a modulator of the expression of genes involved in fatty acid metabolism, it is possible that its increased expression, at least in part contributes to the previously reported accumulation of long-chain acyl-carnitines in fibroblasts [16]. Another study showed that *PGC-1α* overexpression was triggered by VPA in SH-SY5Y neuroblastoma cells and upregulated expression of genes involved in mitochondrial function, glucose transport, fatty acid metabolism and synaptic function [22]. In addition to *PGC-1α*, several other mtDNA maintenance genes and mitochondrial proteins showed increased expression pattern, confirming that, opposite to what we expected, VPA resulted in significantly increased mitochondrial biogenesis, which was further supported by the increased mtDNA copy numbers. These results suggest that *PGC-1α* regulates multiple pathways and that VPA or other HDAC inhibitors may be good candidates to target *PGC-1α* in human disorders. The effect of VPA on mitochondrial biogenesis was observed both in control and patient cell lines, but the capacity of mutant *POLG* to increase the expression of mitochondrial genes and to elevate mtDNA copy numbers was less effective. A similar mechanism in other mitochondrial conditions may explain the inability of the mitochondrial respiration to adjust the demand of a higher respiratory rate triggered by VPA administration. Recently, it has been shown, that valproyl-CoA affects the activity of the succinate CoA ligase (SUCL) and might influence the activity of NDPK inducing an imbalance of nucleotides in the mitochondria [23].

Contrary to our hypothesis that differences in DNA methylation may contribute to *POLG*-mediated VPA toxicity, no evidence of substantive differences in DNA methylation across the genome (as measured using the Illumina HumanMethylation 450 BeadChip, which assays around 1–2% of all CpG sites) was observed between *POLG* mutated patients and controls. We observed VPA-induced shifts of more than 20% in DNA methylation at several thousand CpG sites; however, none surpassed the stringent threshold for genome-wide significance imposed, likely due to the small sample size. The observed differential methylation is consistent with those seen in other contexts including the alteration of DNA methylation in a VPA-treated neuroblastoma cell line [24]. However, there were no discernible differences between the response of controls and *POLG* mutated patients at these apparently VPA-responsive loci. Given the marked perturbation of gene expression observed in the cell lines studied, if DNA methylation were an important mechanism of gene regulation in this context, we would have expected differences between *POLG* and control cell lines to have been observed. We therefore conclude that altered DNA methylation is unlikely to make a major contribution to POLG-mediated VPA toxicity, although it may play a role in other mechanistic pathways culminating in adverse responses to this drug.

In summary, our data provide experimental evidence that VPA triggers increased mitochondrial biogenesis, probably through the increased expression of several genes involved in these mechanisms. We could not show any genome-wide differences in DNA methylation patterns between *POLG* mutated patients and controls; VPA caused similar changes in both patient and control cell lines. However, the capacity of POLG-deficient cells to address the increased metabolic rate caused by VPA administration was significantly impaired. Our data suggest that by exhausting metabolic reserve capacity of the cells it may have a deleterious effect in *POLG*-related disease. By combining these results with our previous data, we can propose the following model to explain VPA-induced liver failure. We suggest that VPA triggers higher metabolic rate and increased function of the mitochondrial respiratory chain; however, genetic defects (e.g. *POLG* mutations) prevent the optimal increase in metabolism, by altering mtDNA replication or repair mechanisms.

Could VPA be beneficial by increasing mitochondrial biogenesis in other forms of mitochondrial disease? Because of the high metabolic rate and increased repair mechanisms in liver, VPA may more readily exhaust endogenous hepatocellular regenerative mechanisms, especially if further compromised with altered mitochondrial accommodation due to mutations in *POLG*. Further studies are needed to decide whether similar mechanisms may be beneficial or rather harmful in other types of mitochondrial disease.

## Funding

RH was supported by the Medical Research Council (UK) (G1000848) and the European Research Council (309548). PFC is a Wellcome Trust Senior Fellow in Clinical Science and a National Institute for Health Research Senior Investigator who also receives funding from the Medical Research Council (UK), the UK Parkinson's Disease Society and the UK National Institute for Health Research Biomedical Research Centre for Ageing and Age-related disease award to the Newcastle upon Tyne Foundation Hospitals NHS Trust. CR and HRE are members of the Medical Research Council Integrative Epidemiology Unit at the University of Bristol, supported by funds from the Medical Research Council. HRE is additionally supported by a research fellowship from the Oak Foundation.

## Conflict of interest

The authors declare no conflict of interests.

## Figures and Tables

**Table 1:** Properties of investigated cell lines derived from five *POLG*-positive patients and three healthy control individuals.

Patient	Age (years)	Sex	cDNA	Consequence	Protein domain affected	Detailed clinical phenotype						
						OA	Epilepsy	Ataxia	Myopathy	Neuropathy	PEO	Others
**P1**	1	M	c.2209 G>C; c.2300 C>A	p.Gly737Arg; p.Ala767Asp	LR	−	+	−	+	N/A	−	AHS
**P2**	5	M	c.1399G>A; c.3311C>T	p.Ala467Thr; p.Se1104Phe	LR; PD	−	+	+	+	N/A	−	AHS
**P3**	3	F	c.2243G>C; c.3600delT	p.Trp748Ser; c.3600delT	LR; PD	−	+	−	+	N/A	−	AHS
**P4**	39	M	c.1399 G>A; c.2243G>C	p.Ala467Thr; p.Trp748Ser	LR	−	−	+	−	+	+	MIRAS
**P5**	36	M	c.2243G>C; c.2243G>C	p.Trp748Ser; p.Trp748Ser	LR	−	−	+	−	+	+	MIRAS
**C1**	47	F	−	−	−	−	−	−	−	−	−	−
**C2**	25	F	−	−	−	−	−	−	−	−	−	−
**C3**	56	F	−	−	−	−	−	−	−	−	−	−

**Table 2:** Analysis of the methylation status of probes selected within the loci of interest, as well as within CpG islands that were flanking each individual locus within 2kb of the distal end of the island. No CpG sites achieved statistical significance following multiple test correction (*P* < 0.05).

Locus	Chromosomal location (UCSC build 37)	Region selected from 450K (region + (CpG Island + 2kb))	Number of analyzed probes (CpG sites)	Absolute change in methylation following VPA treatment		Fold change in methylation following VPA treatment	
				Controls	POLG	Controls	POLG
*PEO1*	chr10:102,747,293-102,754,158	chr10:102,744,534-102,754,158	**23**	− 0.1%	+ 0.1%	1.04	1.03
	CpG island chr10:102,746,534-102,747,501						
*POLG*	chr15:89,859,536-89,878,026	chr15:89,859,536-89,880,597	**2**	− 6.7%	+ 2.7%	0.91	1.08
	CpG island chr15:89,877,598-89,878,597						
*POLG2*	chr17:62,473,902-62,493,184	chr17:62,473,902-62,495,323	**8**	− 1.1%	− 0.7%	1.06	1.04
	CpG island chr17:62,492,883-62,493,323						
*POLRMT*	chr19:617,223-633,568	chr19:613,692-635,678	**30**	+ 2.2%	− 1.5%	1.08	1.04
	CpG island (intragenic) chr19:615,692-623,505						
	CpG island chr19:632,698-633,678						
*PPARG*	chr3:12,329,349-12,475,855	chr3:12,327,428-12,475,855	**21**	− 1.7%	− 1.3%	1.07	1.04
	CpG island chr3:12,329,428-12,330,333						
*PGC1-α*	chr4:23,793,644-23,891,700	chr4:23,793,644-23,891,700	**11**	+ 1.9%	+ 3.1%	1.11	1.08
	No local CpG islands						
*TFAM*	chr10:60,144,903-60,158,990	chr10:60,142,724-60,158,990	**13**	− 0.3%	+ 0.1%	1.03	1.04
	CpG island chr10:60,144,724-60,145,335						

**Table 3:** Quantification of (i) all “top hit” CpG sites (i.e., those ones where methylation changed of more of 20% and/or 2-fold) as well as (ii) mitochondria, (iii) liver and (iv) both mitochondria and liver-associated “top hit” CpG sites, in controls and *POLG*-affected patients. The statistical comparisons between controls and patients were performed using two-tailed chi-square test with Yates' correction. None of the group comparison appeared to be statistically significant.

	Controls	*POLG* patients	Controls vs. patients (*P*-values)
All “top hit” CpG sites	6898	7891	–
Mitochondria specific “top hit” CpG sites	263	314	0.632
Liver-associated “top hit” CpG sites	33	29	0.361
Mitochondria and liver-associated “top hit” CpG sites	4	3	0.859
